# Glycolysis Dependency as a Hallmark of *SF3B1*-Mutated Cells

**DOI:** 10.3390/cancers14092113

**Published:** 2022-04-24

**Authors:** Raquel Vivet-Noguer, Malcy Tarin, Christine Canbezdi, Stephane Dayot, Lisseth Silva, Alexandre Houy, Sylvain Martineau, Virginie Mieulet, Géraldine Gentric, Damarys Loew, Bérangère Lombard, Fariba Nemati, Sophie Richon, Lea Guyonnet, Vincent Servois, Stephan Vagner, Marc-Henri Stern, Sergio Roman-Roman, Samar Alsafadi

**Affiliations:** 1 Translational Research Department, Institut Curie, PSL Research University, 75248 Paris, France; raquelvivetnoguer@gmail.com (R.V.-N.); malcy.tarin@curie.fr (M.T.); christine.canbezdi@curie.fr (C.C.); lisseth.silva@curie.fr (L.S.); fariba.nemati@curie.fr (F.N.); sergio.roman-roman@curie.fr (S.R.-R.); 2 INSERM U830, DNA Repair and Uveal Melanoma (D.R.U.M.), Equipe labellisée par la Ligue Nationale Contre le Cancer, Institut Curie, PSL Research University, 75248 Paris, France; stephane.dayot@gustaveroussy.fr (S.D.); alexandre.houy@curie.fr (A.H.); marc-henri.stern@curie.fr (M.-H.S.); 3 INSERM U1279, Gustave Roussy Institute, Paris-Saclay University, 94800 Villejuif, France; 4 CNRS UMR3348, INSERM U1278, Institut Curie, PSL Research University, 75248 Paris, France; sylvain.martineau@curie.fr (S.M.); stephan.vagner@curie.fr (S.V.); 5 CNRS UMR3348, INSERM U1278, Université Paris-Saclay, 91190 Gif-sur-Yvette, France; 6 INSERM U830, Stress and Cancer Laboratory, Equipe labellisée par la Ligue Nationale Contre le Cancer, Institut Curie, PSL Research University, 75248 Paris, France; virginie.mieulet@inserm.fr (V.M.); geraldine.gentric@curie.fr (G.G.); 7 Mass Spectrometry and Proteomics Facility, Institut Curie, PSL Research University, 75248 Paris, France; damarys.loew@curie.fr (D.L.); berangere.lombard@curie.fr (B.L.); 8 CNRS UMR 144, Institut Curie, PSL Research University, 75248 Paris, France; sophie.richon@curie.fr; 9 Cytometry Core, Institut Curie, PSL Research University, 75248 Paris, France; lea.guyonnet@curie.fr; 10 Department of Radiology, Institut Curie, PSL Research University, 75248 Paris, France; vincent.servois@curie.fr

**Keywords:** glycolysis, metabolism, SF3B1, splicing, uveal melanoma

## Abstract

**Simple Summary:**

Cancer-associated *SF3B1* mutations result in aberrant transcripts whose fate remains unknown. We aimed to investigate the functional consequences of these splice aberrations. Our results show that *SF3B1* mutation alters the translation of transcripts encoding proteins involved in metabolism, which triggers a metabolic switch toward an increased glucose uptake. Consequently, *SF3B1*-mutated cells are more sensitive to glycolysis inhibition than *SF3B1* wild-type cells.

**Abstract:**

*SF3B1* mutations are recurrent in cancer and result in aberrant splicing of a previously defined set of genes. Here, we investigated the fate of aberrant transcripts induced by mutant SF3B1 and the related functional consequences. We first demonstrate that mutant SF3B1 does not alter global nascent protein synthesis, suggesting target-dependent consequences. Polysome profiling revealed that 35% of aberrantly spliced transcripts are more translated than their corresponding canonically spliced transcripts. This mostly occurs in genes with enriched metabolic functions. Furthermore, LC-MS/MS analysis showed that mutant SF3B1 impacts the abundance of proteins involved in metabolism. Functional metabolic characterization revealed that mutant SF3B1 decreases mitochondrial respiration and promotes glycolysis to compensate for defective mitochondrial metabolism. Hence, mutant SF3B1 induces glycolysis dependency, which sensitizes cells to glycolysis inhibition. Overall, we provide evidence of the oncogenic involvement of mutant SF3B1 in uveal melanoma through a metabolic switch to glycolysis, revealing vulnerability to glycolysis inhibitors as a promising therapeutic strategy.

## 1. Introduction

Splicing defects are recurrent in cancer and are mostly caused by mutations of genes encoding for splicing factors involved in 3′ splice site (3′ss) recognition. *SF3B1* (Splicing Factor 3b, Subunit 1) is the most frequently mutated splicing gene in cancer, recurrently found in hematological malignancies (28% in myelodysplastic syndromes, 15% in chronic lymphocytic leukemia) and 23% of uveal melanoma (UM), the most common primary intraocular tumor in adults [1,2,3,4,5]. Of note, UM is driven by activating mutations in Gαq pathway, which are recurrently associated with a second mutation in *BAP1* (BRCA1-associated protein 1), *SF3B1* or *EIF1AX* (eukaryotic translation initiation factor 1A X-linked). Several *SF3B1* hotspot mutations have been described at the C-terminal of the HEAT (Huntingtin, Elongation factor 3, protein phosphatase 2A, Targets of rapamycin 1) repeats with a different prevalence according to the cancer type. Notably, mutations targeting codon R625 predominate in UM [3,6,7]. Thus, *SF3B1* mutations are change-of-function missense mutations that lead to the recognition of cryptic 3′ss, and thereby to aberrant splice junctions in a specific set of transcripts. These splicing aberrations have been thoroughly characterized by our group and others in recent years [7,8,9,10]. It is now well established that mutant SF3B1 corrupts the recognition of the intronic branchpoint sequence by the U2 snRNP complex and promotes the recognition of a cryptic branchpoint located upstream of the canonical site, thereby leading to the inclusion of an intronic sequence in the final mRNA product.

Although splicing aberrations induced by *SF3B1* mutations are now well defined, little is known about the fate of the resulting aberrant transcripts. Recent studies have brought to light the different functional impacts of these aberrant transcripts. For instance, it has been demonstrated that *SF3B1* mutations lead to the production of an aberrant splicing isoform of *MAP3K7* pre-mRNA containing a premature codon triggering its degradation by NMD (Nonsense-Mediated mRNA Decay). This defect in MAP3K7 production in turn leads to hyperactivation of the NF-κB pathway that is known to drive MDS (myelodysplastic syndromes) [11,12]. *SF3B1* mutations have also been reported to induce the inclusion of a poison exon in *BRD9* mRNA, leading to BRD9 repression. Interestingly, correcting *BRD9* missplicing by antisense oligonucleotides or mutagenesis repressed tumor growth [13]. Similarly, *SF3B1*^K700E^-induced missplicing of *PPP2R5A* leads to its downregulation and to a concomitant increase in the stability of the MYC protein, thereby altering apoptosis [14]. More recently, SF3B1 mutations have been shown to lead to *DVL2* missplicing, a gene that encodes a negative regulator of the Notch pathway [15,16]. These missplicing-driven dysregulations of specific targets can then contribute to the oncogenic role of *SF3B1* mutations, yet the impact of *SF3B1* mutations seems to exceed these specific alterations.

Interestingly, mutant SF3B1 has been shown to alter the abundance of proteins affecting metabolic pathways in breast cancer cells [17]. Proteins enriched in the mitochondrial electron transport chain (ETC), including UQCC1 (ubiquinol-cytochrome C reductase complex assembly factor 1), whose pre-mRNA is aberrantly spliced by mutant SF3B1, showed reduced abundance. *SF3B1*^K700E^ cells were shown to present a reduced mitochondrial respiration capacity, while the ECAR (extracellular acidification rate) was unaltered. *PHGDH*, encoding for an enzyme involved in the de novo synthesis of serine, is also aberrantly spliced by mutant SF3B1. Consequently, *SF3B1*^K700E^ cells are more sensitive to serine and glycine starvation than *SF3B1*^WT^ cells [17]. Prior studies linked defects in splicing factors to metabolic impairment. For example, loss of expression of *SRSF3* (Serine/arginine-Rich Splicing Factor 3) triggers the missplicing of key regulators of glucose and lipid metabolism in hepatocytes [18]. In breast cancer cells, knockdown of *ESRP1* (Epithelial Splicing Regulatory Protein 1) decreased the expression of fatty acid, lipid metabolism targets and oxidoreductases including *PHGDH* (D-3-phosphoglycerate dehydrogenase). *ESPR1* depletion increased basal and spare respiration capacity, while no changes were seen in the ECAR [19]. Co-occurrence of *SF3B1*^R625^ with *NF1*, *KIT* or *KRAS* mutations, affecting metabolism, has been reported in other forms of melanoma but not in uveal melanoma [20,21,22].

Overall, growing evidence argues for therapeutic targeting of metabolism in cells with splicing defects, providing therapeutic alternatives to splicing inhibitors that lack specificity and confer cytotoxic effects [4].

Here, we explored the fate of aberrant transcripts induced by mutant SF3B1 and the related functional consequences. Our work provides evidence that *SF3B1* mutation alters the translation of specific transcripts encoding proteins involved in metabolism, which triggers a metabolic switch toward an increased glucose uptake. Consequently, *SF3B1*-mutated cells were more sensitive to glycolysis inhibition than *SF3B1* wild-type cells. Thus, our study sheds light on a new aspect of the oncogenic impact of mutant SF3B1 and provides an appealing therapeutic approach based on glycolysis inhibition, possibly combined with targeting other cancer-related pathways.

## 2. Materials and Methods

Cell model development and maintenance. The Mel202 cell line was obtained from the European Searchable Tumour Line Database (CVCL_C301, Tubingen, University, Germany). A destabilizing domain (DD) tag derived from human FKBP12 was inserted by CRISPR/Cas9 at the endogenous R625G-SF3B1 protein in Mel202, following the previously reported Degron-KI system [23]. Both *SF3B1*^WT^ and *SF3B1*^R625G^ Mel202 cells were cultured with RPMI-1640 supplemented with 10% fetal bovine serum (FBS). A point mutation in *SF3B1* leading to a G742D amino-acid substitution was inserted in the HAP1 cell line using the Lipofectamine^TM^ CRISPRMAX^TM^ Cas9 Transfection Reagent (CMAX00008, Invitrogen) following the manufacturer’s instructions. The isogenic HEK293T cell model harboring *SF3B1*^K666T^ was generated by CRISPR/Cas9 as previously described [7]. The HAP1 and HEK293T cell lines were cultured with DMEM supplemented with 10% FBS. The mutational status of all cell lines was verified with Sanger sequencing and RNA-seq.

Analysis of nascent protein synthesis. The cells were incubated with a methionine-free RPMI medium supplemented with 2% FBS for 1 h. Then, 50 µM of Click-IT AHA Reagent (L-Azidohomoalanine from Invitrogen C101102) was added and incubated for 4 h. Cells were then washed three times with PBS, lysed with 1% SDS in 50 mM Tris-HCl (pH 8), incubated for 30 min in ice and then sonicated. Equivalent amounts of protein lysates underwent Click-It reaction using the kit (Click-iT^TM^ Protein Reaction Buffer Kit Invitrogen C10276) according to the manufacturer’s protocol. Biotinylated nascent proteins were immunoblotted against anti-biotin and β-actin (Cell Signaling #5597 and #3700 respectively) antibodies.

Isolation of polysome fractions, RNA extraction and sequencing. Cells were plated in 15-cm Petri dishes and incubated overnight for a 60–70% confluency. A total number of 30 million cells were then treated with 100 µg/mL of cycloheximide (CHX) and incubated at 37 °C, 5% CO_2_ for 15 min. Cells were washed with PBS containing CHX and lysed with a lysis buffer containing 20 mM Tris pH 7.5, 100 mM NaCl, 3 mM MgCl2 and fresh 1 mM DTT, 100 U/mL RNAsine and 100 µg/mL CHX. Cell lysates were centrifuged and loaded onto a sucrose gradient of 15–50% and ultracentrifuged in a rotor SW41 Ti. The resulting samples were then separated by a fractioning system (Brandel/Teledyne ISCO) equipped with a spectrophotometer to monitor absorbance at 260 nm. RNA was extracted from polysomal fractions with a phenol:guanidine isothiocyanate monophasic solution with TRIZOL LS following standard protocols. For polysome sequencing, two replicates were sequenced for each condition. Monosome-like samples consisted of RNA extracted from fractions 2–8, while polysome-like samples included RNA extracted from fractions 9–14.

RNA-seq analysis. Libraries were constructed using the TruSeq Stranded mRNA Sample Preparation Kit (Illumina) and sequenced on an Illumina NovaSeq platform using a 100-bp paired-end sequencing strategy. High depth RNA-sequencing data (from 96M to 126M reads) were mapped using STAR (v 2.7.0e) and expression of junctions from aberrant splicing signatures described by our group and others [7,8] were used to perform hierarchical clustering with R.

Quantitative mass spectrometry analysis. Cells were washed with PBS and incubated with a lysis buffer supplemented with phosphatase and protease inhibitors for 45 min in ice. Upon centrifugation, the supernatants were solubilized in 8 M urea and 50 mM NH4HCO3. After dilution to a final concentration of 1M urea, proteins were reduced, alkylated and digested overnight prior to liquid chromatography-tandem mass spectrometry analysis of triplicates, mainly as previously described [24]. Data were further processed using myProMS v3.9.1 [25] and protein with at least three total peptides in all replicates, an absolute 1.2-fold enrichment and an adjusted *p*-value ≤ 0.05 was considered significantly dysregulated in the sample comparison. Unique proteins were considered with at least three total peptides in all replicates. Mass spectrometry proteomics data were deposited in the ProteomeXchange Consortium via the PRIDE [26] partner repository with the dataset identifier PXD022726.

Immunoblot analysis. Cells were lysed in RIPA buffer and proteins were quantified using a BCA Protein Assay (Pierce). Equal amounts were separated by SDS–polyacrylamide gel electrophoresis, transferred to nitrocellulose membranes, followed by immunoblotting with specific primary antibodies for OXPHOS cocktail (ab110411; Abcam), PDH1 (E1 subunit; ab10330; Abcam), G6PD (CST12263, Cell Signaling), PHGDH (HPA021241; Sigma), Phospho-PDH (E1 subunit)—S293 (ab177461; Abcam). The membrane was then incubated with anti-rabbit or anti-mouse Odyssey secondary antibodies. Immunolabelled proteins were detected using the Odyssey Infrared Imaging System (Li-cor). β-actin immunoblotting was used to quantify and normalize the results.

Measurement of oxygen consumption rate (OCR) and extracellular acidification rate (ECAR). Cells were plated in Seahorse XF96 Analyzer plates and incubated overnight at 37 °C with 5% CO_2_. Cells were washed and media was replaced by unbuffered assay media at pH 7.4 for ECAR analysis, and unbuffered assay media at pH 7.4 containing 25 mM of glucose, 4 mM of glycine and 1 mM of pyruvate for OCR measurement. The cells were then incubated at 37 °C without CO_2_ for 1 h. Cells were injected with 1 µM (for HAP1) or 2 µM (for Mel202 and HEK293T) oligomycin, 2 µM carbonyl cyanide-p-trifluoromethoxyphenylhydrazone (FCCP) and 2 µM rotenone/antimycin A. Wave 2.6 software was used for the analysis.

Mitochondrial content and ROS levels analysis. Cell mitochondrial content and ROS levels were assessed by staining with Mitotracker Red FM (Molecular Probes MM22425) and CellROX Green Reagent (Molecular Probes C10444), respectively. Cells were incubated with either 250 nM of Mitotracker Red FM or 2 µM of CellROX Green Reagent for 30 min at 37 °C. Cells were then washed with PBS, trypsinized and resuspended with a buffer of PBS with 2% FBS and 2mM EDTA for flow cytometric analysis. Flow cytometry data were acquired using a ZE5 cell analyzer (BioRad) and analyzed with FlowJo analysis software (FlowJo, LLC).

Viability assay. Cells were treated with increasing concentrations of PFK158 (Selleckchem S8807) (0–5 µM) for 48 and 72 h. Cell viability was assessed by Orangu *colorimetric* assay according to the manufacturer’s instructions (OR01-500, Cell Guidance Systems) and incubated for 3 h. Absorbance at 450 nm was read using a TECAN-Spark microplate reader.

Glucose uptake assay. Glucose uptake was measured in cells following the manufacturer’s recommendations (Glucose Uptake-Glo^TM^ Assay Promega J1342). The cells were starved for glucose by preincubation with Krebs-Ringer-Phosphate-HEPES buffer containing 2% BSA for 40 min and washed with PBS. Next, 50 µL of 1 mM 2DG was added and incubated at RT for 20 min. After the addition of Stop Buffer and Neutralization Buffer, 2DG6P Detection Reagent was added and incubated for 2 h. Luminescence was recorded at 0.5-s integration using a TECAN-Spark microplate reader.

## 3. Results

### 3.1. Translated SF3B1^R625G^-Induced Aberrantly Spliced Transcripts Are Enriched in Metabolic Pathways

To validate our isogenic model of Mel202-*SF3B1*^R625G^ and CRISPR/Cas9-generated Mel202-*SF3B1*^WT^ cells, we analyzed the aberrant splicing pattern in *SF3B1*^R625G^ cells by RNA sequencing (RNA-seq). In fact, 1445 aberrant splice junctions of the previously established splice pattern in *SF3B1*-mutated tumors [7,8] were found to be differentially expressed in *SF3B1*^R625G^ cells as compared to *SF3B1*^WT^ cells (Supplementary Figure S1A,B, Supplementary Table S1).

**Figure 1:**
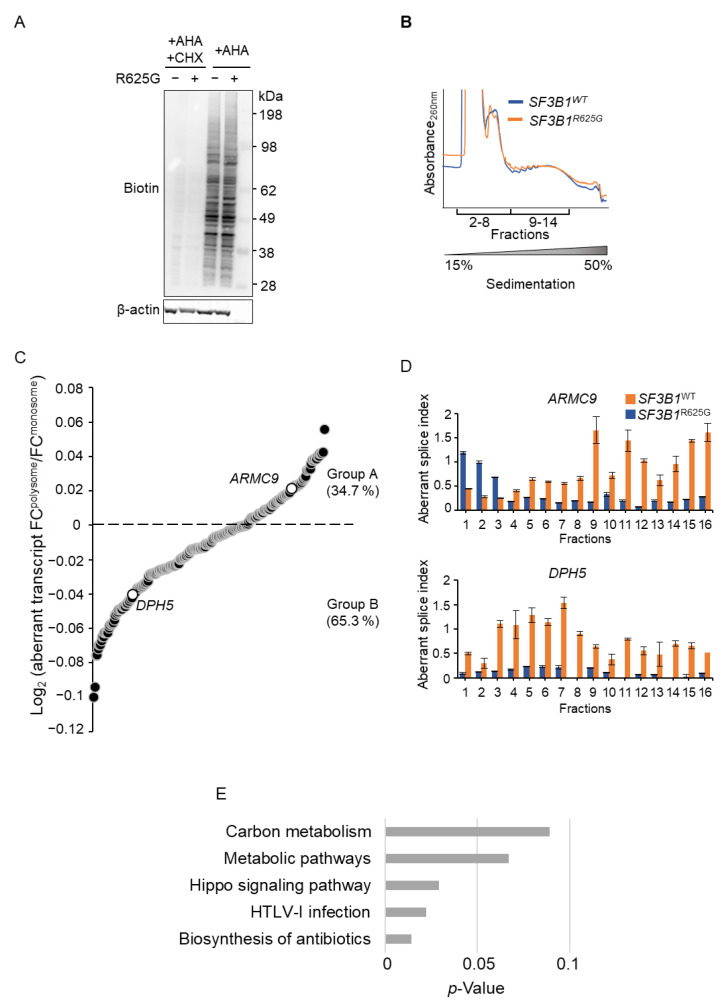
Impact of *SF3B1*^R625G^ on nascent protein synthesis and translation fate of the *SF3B1*^R625G^-induced splicing transcripts. (**A**) Nascent protein synthesis in *SF3B1*^WT^ and *SF3B1*^R625G^ Mel202 cells by Click-iT L-azidohomoalanine (AHA) labeling. The cells were starved of methionine for 1 h and incubated with AHA for 3 h. Lysates underwent a Click-iT reaction in which azide tags reacted with alkyne-biotin and biotin was visualized by immunoblotting. Cycloheximide-treated (CHX) cell lysates were used as a control for protein synthesis inhibition. β-actin was immunoblotted as a control for protein quantity. (**B**) Polysome profiles of the *SF3B1*^WT^ (blue) and *SF3B1*^R625G^ (orange) Mel202 cells. The sequenced samples are highlighted (pooled monosome fractions 2–8; and pooled polysome fractions 9–14). The sucrose gradient 15–50% of sample fractioning is displayed. (**C**) The log_2_FC(normalized expression in polysome fractions/ normalized expression in monosome fractions) of the *SF3B1*^R625G^ cells as determined by RNA-seq is plotted for each aberrantly spliced transcript (*p*-values ≤ 10^−5^). The aberrantly spliced transcripts with positive log_2_FC values (group A) are relatively more translated than their corresponding canonically spliced transcripts, while those with negative log_2_FC values (group B) are less translated than their corresponding canonically spliced transcripts. *ARMC9* and *DPH5* are highlighted. (**D**) The aberrant splice index (aberrantly spliced transcript/canonical transcript) obtained by RT-qPCR for the *SF3B1*^R625G^-sensitive genes *DPH5* and *ARMC9* is plotted for each fraction from *SF3B1*^WT^ (blue) and *SF3B1*^R625G^ (orange) Mel202 cells. (**E**) KEGG pathway enrichment analysis of the translated aberrantly spliced transcripts (group A) identified by polysome profiling (Figure 1C). The enriched KEGG pathways are represented against the minus log *p*-value. Only significant splice transcripts are displayed (*p*-value ≤ 10^−5^).

We then investigated whether the *SF3B1*^R625G^ affects the translatome, provided that certain splicing factors also act as regulators of translation [27,28,29,30,31]. We labeled the protein lysates of *SF3B1*^WT^ and *SF3B1*^R625G^ Mel202 cells with L-azidohomoalanine (AHA) and performed a click reaction with alkyne-biotin in order to assess the global levels of nascent protein synthesis. While cycloheximide (CHX) treatment efficiently inhibited protein synthesis, *SF3B1*^WT^ and *SF3B1*^R625G^ cells displayed comparable signals of nascent protein synthesis. This finding implies that *SF3B1*^R625G^ does not impact global protein synthesis (Figure 1A and Figure S2A).

To assess the impact of *SF3B1* mutation on the translatome at a transcript-specific level, we next investigated whether *SF3B1*^R625G^-induced aberrantly spliced mRNAs are efficiently translated into proteins. We performed polysome profiling, followed by RNA-seq, in *SF3B1*^WT^ and *SF3B1*^R625G^ cells. We observed comparable polysome profiles of *SF3B1*^WT^ and *SF3B1*^R625G^ cells (Figure 1B), suggesting that *SF3B1*^R625G^ does not impact global translation efficiency.

Figure 1C shows the fractional distribution of the *SF3B1*^R625G^-aberrant transcripts plotted as the logarithmic ratio of each aberrant transcript fold change in polysomes to its fold change in monosomes in *SF3B1*^R625G^ cells. Our results indicate that 35% of aberrantly spliced transcripts in *SF3B1*^R625G^ cells are relatively more translated than their corresponding canonically spliced transcripts (group A), while 65% are less translated (group B) (Figure 1C and Supplementary Table S2). To validate and illustrate this finding, we analyzed the translation efficiency of the aberrantly spliced transcripts of *ARMC9* (from group A) and *DPH5* (from group B). We performed RT-qPCR analysis in each of the fractions to determine the relative abundance of the aberrant transcript to the canonical transcript, which we called the aberrant splice index. As expected, the aberrant splice index increased for both *ARMC9* and *DPH5* in the *SF3B1*^R625G^ cells as compared to the *SF3B1*^WT^ cells. Notably, in *SF3B1*^R625G^ cells, the *ARMC9* aberrant splice index was higher in fractions 9–16 (containing mRNAs that are engaged in polysomes) than in other fractions, indicating that the *ARMC9* aberrant transcript is relatively more translated than the canonical *ARMC9* transcript in *SF3B1*^R625G^ cells. In contrast, the *DPH5* aberrant splice index was higher in fractions 3–8 (containing mRNAs that are not engaged in polysomes) than in other fractions (Figure 1D). This suggests that the *DPH5* aberrant transcript is relatively less translated than the canonical *DPH5* transcript in *SF3B1*^R625G^ cells. Thus, our results imply the transcript-dependent fate of *SF3B1*^R625G^-induced aberrant transcripts.

Next, we focused on the subset of genes for which the aberrantly spliced transcripts in *SF3B1*^R625G^ cells are relatively more translated than their corresponding canonically spliced transcripts (group A) to screen for potential oncogenic functions. Enrichment analysis of the KEGG pathways revealed a major involvement of group A in metabolism, including carbon metabolism and metabolic pathways (Figure 1E and Supplementary Table S3).

Conclusively, our findings show that the *SF3B1* mutation impacts translation in a transcript-dependent manner. While most *SF3B1*^R625G^-induced aberrantly spliced transcripts are less translated than their corresponding canonically spliced transcripts (group B, 65%), those that are more translated (group A, 35%) tend to encode proteins involved in metabolism.

### 3.2. SF3B1^R625G^ Impacts the Abundance of Proteins Involved in Metabolism

**Figure 2:**
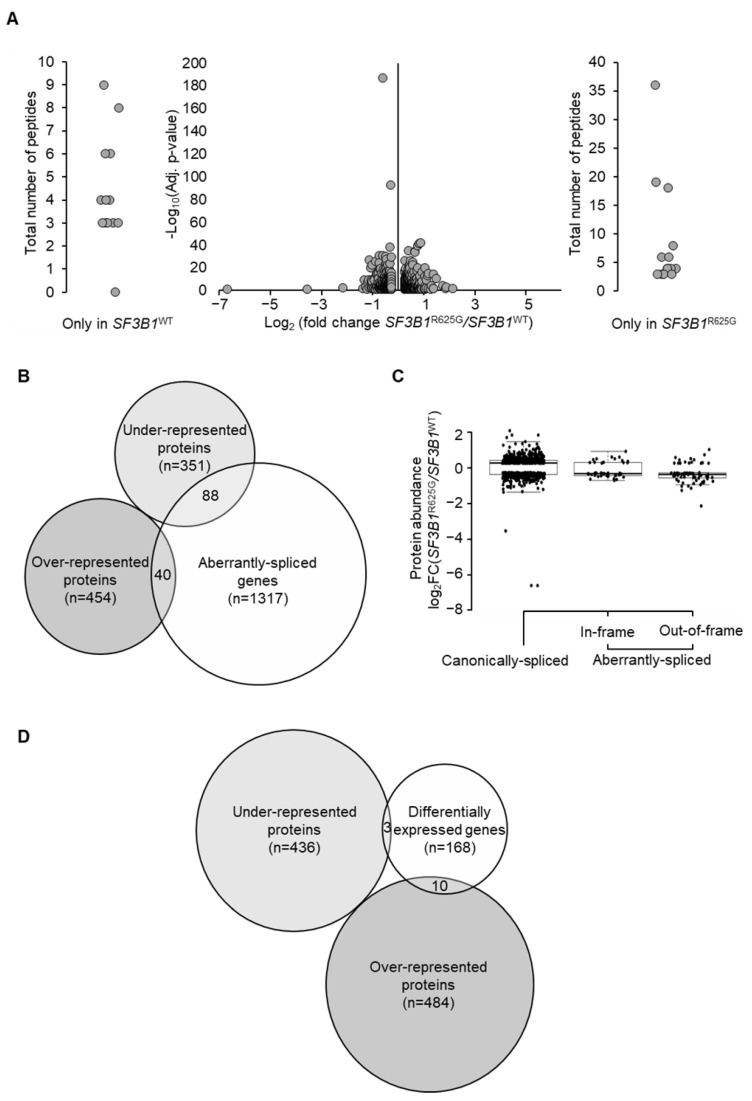
*SF3B1*^R625G^ impact on protein abundance. (**A**) Differential protein abundance analysis obtained using quantitative label-free LC-MS/MS analysis. The volcano plot illustrates differentially abundant proteins. The minus log *p*-value is plotted against the log_2_FC of protein expression (*SF3B1*^R625G^ *versus* *SF3B1*^WT^). Proteins with at least 3 detected peptides in all replicates (*n* = 3) are displayed; absolute fold change ≥1.2 and *p*-value ≤0.05 are considered as significantly dysregulated. External plots show proteins with peptides identified in only one condition (left in *SF3B1*^WT^ and right in *SF3B1*^R625G^). (**B**) Venn diagram showing a restricted overlap between the differentially abundant proteins (underrepresented and overrepresented) and the aberrantly spliced genes. A total of 8.8% aberrantly spliced genes (*n* = 128/1445) display dysregulated protein levels (*n* = 88 underrepresented proteins and *n* = 40 overrepresented proteins). (**C**) Log_2_FC of protein expression (*SF3B1*^R625G^ *versus* *SF3B1*^WT^) is plotted on the x-axis according to the splice category: canonically spliced genes (genes with no splice aberrations), genes with predicted NMD-sensitive aberrant splice junctions, and genes with predicted NMD-insensitive aberrant splice junctions. (**D**) Overlap between differentially abundant proteins and differentially expressed genes shown by Venn diagram. A total of 7.2% of differentially abundant proteins are encoded by differentially expressed genes (*n* = 13/181; *n* = 3 underrepresented and *n* = 10 overrepresented).

To address the impact of *SF3B1* mutation on protein abundance, we performed a quantitative label-free tandem liquid chromatography-mass spectrometry (LC-MS/MS) analysis of *SF3B1*^WT^ and *SF3B1*^R625G^ Mel202 cells. We quantified a total number of 8573 proteins. As shown in Figure 2A, we found 439 underrepresented and 494 overrepresented proteins in *SF3B1*^R625G^ cells as compared to *SF3B1*^WT^ cells (with at least 3 detected peptides per protein in all replicates, absolute fold change ≥1.2, *p*-value ≤0.05) (Figure 2A and Supplementary Table S4). We then explored genes that are both aberrantly spliced by *SF3B1*^R625G^ and coding for dysregulated proteins according to our quantitative LC-MS/MS findings. Our analysis revealed that only 8.8% of aberrantly spliced genes were associated with dysregulated protein levels, accounting for 2.7% of the overrepresented proteins and 6.1% of the underrepresented proteins (Figure 2B). The *SF3B1*^R625G^-induced splicing aberrations altering protein abundance involved either out-of-frame (*n* = 69) or in-frame junctions (*n* = 44) (Figure 2C). We also compared the differentially expressed genes with the differentially abundant proteins and found that 7.2% of differentially expressed genes encode proteins that are dysregulated by *SF3B1*^R625G^ (5.5% genes encoding overrepresented proteins and 1.7% genes encoding underrepresented proteins) (Figure 2D). Accordingly, the impact of *SF3B1*^R625G^ on the proteome seems to be independent of mRNA abundance.

We then studied the functional impact of proteins dysregulated by *SF3B1*^R625G^ by gene ontology analysis. Enrichment analysis of biological processes revealed a significant underrepresentation of metabolic proteins, implying a metabolic alteration induced by mutant SF3B1. Interestingly, 19 underrepresented metabolic proteins were encoded by genes that are aberrantly spliced by *SF3B1*^R625G^ (*UQCC1, DLST, OGDHL, CBS, AMDHD2, PHGDH, FDPS, NADSYN1, MUT, COASY, POLR1E, GALT, NDUFB1, PPOX, UBA1, PRKDC, PPP2R5A, PPP2R3A,* and *SOAT1*). Notably, proteins involved in mitochondrial respiratory chain complex III assembly were significantly underrepresented, including the main complex III assembly factors UQCC1 and UQCC2. PHGDH, which catalyzes the first step of the L-serine biosynthesis pathway, was also aberrantly spliced and underrepresented (Supplementary Figure S3 and Supplementary Table S5).

### 3.3. SF3B1 Mutation Leads to a Decrease in Mitochondrial Respiration and Promotes Cell Dependency on Glycolysis

**Figure 3:**
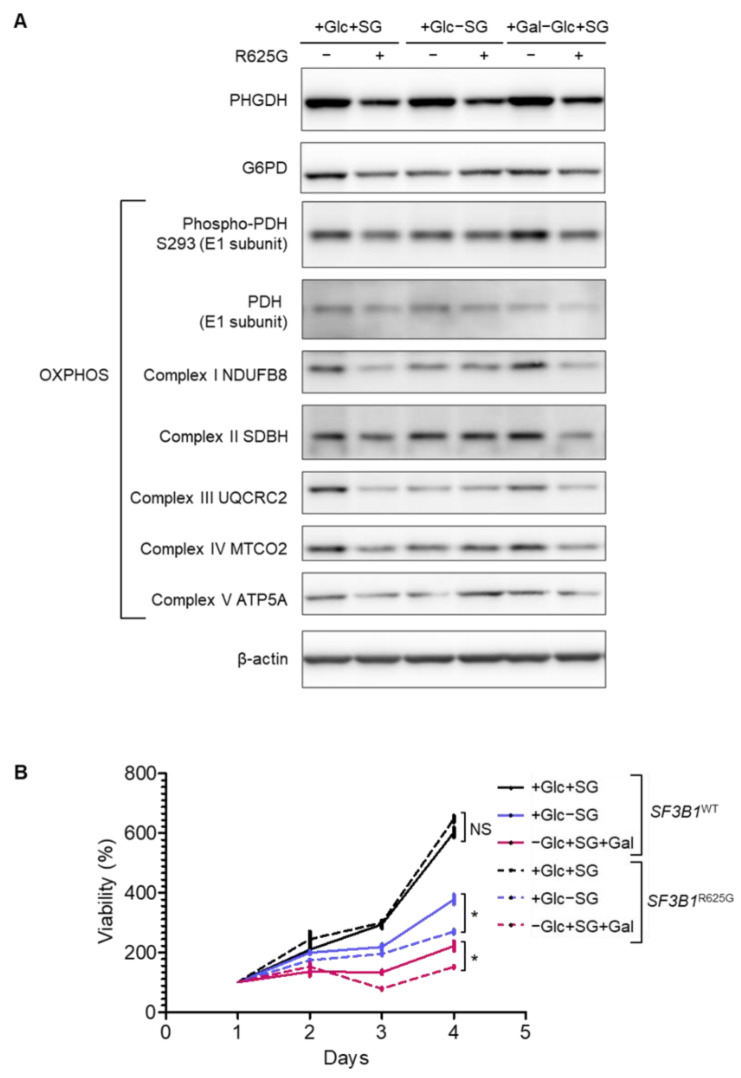
*SF3B1*^R625G^ leads to a decreased expression of metabolic proteins and sensitizes cells to serine, glycine and glucose deprivation. (**A**) Expression of metabolic targets involved in serine de novo synthesis, PPP (pentose phosphate pathway), glycolysis and OXPHOS (oxidative phosphorylation) upon 3 days of media deprivation in *SF3B1*^WT^ and *SF3B1*^R625G^ isogenic Mel202 cells by Western blot. β-actin was immunoblotted as a control for protein quantity. (**B**) Cell viability of *SF3B1*^WT^ and *SF3B1*^R625G^ Mel202 cells upon media starvation (Gal: galactose, Glc: glucose, and SG: serine and glycine). The complete media condition was also included (+Glc+SG). Data are represented as the mean of triplicates ± SD. The Mann–Whitney U test was used to generate the *p*-values; * *p* < 0.05; NS: non-significant.

Considering the *SF3B1*^R625G^-induced dysregulation of proteins involved in metabolism, we conducted a functional evaluation of the metabolic features in *SF3B1*^WT^ and *SF3B1*^R625G^ Mel202 cells. As shown in Figure 3A and Supplementary Figures S2B and S4, *SF3B1*^R625G^ cells display a low basal protein expression of PHGDH compared to *SF3B1*^WT^ cells, which is in line with the LC-MS/MS findings. PHGDH is essential for generating carbon units from SG (serine and glycine). When deprived of SG for 4 days, *SF3B1*^WT^ and *SF3B1*^R625G^ cells display a viability decrease of 37% and 58%, respectively, indicating a higher dependency of *SF3B1*^R625G^ cells on SG as compared to *SF3B1*^WT^ cells (Figure 3B).

Additionally, we show that G6PD (Glucose-6-phosphate dehydrogenase), the key enzyme catalyzing the first step in the oxidative branch of the pentose phosphate pathway (PPP), is decreased in *SF3B1*^R625G^ as compared to *SF3B1*^WT^ cells. This finding is consistent with the underrepresentation of PGD (6-phosphogluconate dehydrogenase), as detected by LC-MS/MS. PGD catalyzes the formation of ribulose 5-phosphate that can be converted into ribose 5-phosphate and further used for nucleotide synthesis. Thus, the underexpression of the initial PPP enzyme G6PD together with the underrepresentation of the enzyme PGD imply a decreased rate of PPP in *SF3B1*^R625G^ cells. Although PPP is one of the main sources of NADPH used to scavenge ROS (Reactive Oxygen Species) [32], we found comparable ROS levels in *SF3B1*^WT^ and *SF3B1*^R625G^ cells (Supplementary Figure S5A,B).

PDH (Pyruvate Dehydrogenase) catalyzes the conversion of pyruvate to acetyl CoA, thus connecting the citric acid cycle and subsequent oxidative phosphorylation (OXPHOS) to glycolysis, gluconeogenesis and lipid and amino acid metabolism pathways. Our results show that the amount of phosphorylated PDH levels is reduced in *SF3B1*^R625G^ cells as compared to *SF3B1*^WT^ cells, suggesting functional impairment of PDH in *SF3B1*^R625G^ cells (Figure 3A).

In line with our findings on *SF3B1*^R625G^-induced missplicing of UQCC1 and UQCC2 and low abundance of OXPHOS proteins, immunoblotting analysis showed decreased expression of OXPHOS complexes in *SF3B1*^R625G^ cells as compared to *SF3B1*^WT^ cells. A further decrease in the expression of complexes I, III, IV and V was observed in *SF3B1*^WT^ cells after SG deprivation, while the expression was maintained in *SF3B1*^R625G^ cells (Figure 3A and Supplementary Figures S3 and S4).

Considering these mitochondrial defects in *SF3B1*^R625G^ cells, we investigated whether cell reliance on OXPHOS to generate ATP increased in *SF3B1*^R625G^ cells. Cells grown in media rich in glucose rely on glycolysis and are less affected by mitochondrial defects. We therefore replaced glucose (Glc) with galactose (Gal) in cell culture media to increase cell reliance on OXPHOS to meet the energy demand. At 4 days, the growth of *SF3B1*^WT^ and *SF3B1*^R625G^ cells decreased by 63% and 77%, respectively, implying that *SF3B1*^R625G^ accentuates the cell reliance on glycolysis, probably due to their defective mitochondrial respiration (Figure 3B).

To further comprehend the observed glycolysis dependency in *SF3B1*^R625G^ cells, we assessed mitochondrial respiration and glycolysis by measuring the OCR (oxygen consumption rate) and ECAR of *SF3B1*^WT^ and *SF3B1*^R625G^ cells by Seahorse XF Analyzer [33]. Basal respiration was calculated by subtracting non-mitochondrial respiration from the first OCR measurement. Next, oligomycin, an inhibitor of ATP synthase, is added and the resulting value is subtracted from the total cellular oxygen consumption to determine ATP-linked respiration. Non-mitochondrial respiration can be subtracted to obtain proton-leak respiration. Then, FCCP (carbonyl cyanide-p-trifluoromethoxyphenyl-hydrazon) is added. FCCP collapses the inner membrane gradient by making the inner membrane permeable to protons. This drives the ETC to operate at its maximum rate. The value obtained by subtracting non-mitochondrial respiration from this quantity represents the maximum respiratory capacity. Finally, antimycin A, a complex III inhibitor, and rotenone, a complex I inhibitor, are added to shut down mitochondrial respiration. The resulting measurement represents non-mitochondrial respiration, a measurement that can be used with other measurements to calculate respiratory parameters. Reserve capacity is calculated by subtracting basal respiration from maximum respiratory capacity [33].

**Figure 4:**
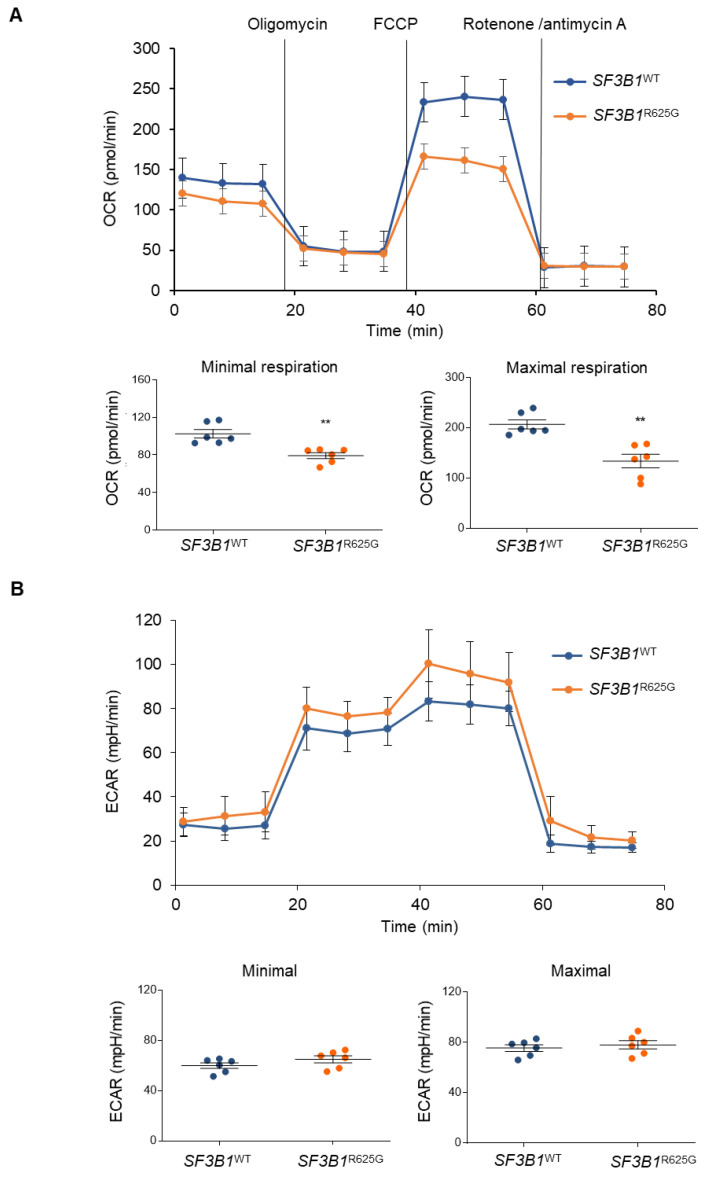
*SF3B1*^R625G^ triggers a decrease in mitochondrial respiration. (**A**) Oxygen consumption rate (OCR) of *SF3B1*^WT^ (blue) and *SF3B1*^R625G^ (orange) isogenic Mel202 cells as measured by Seahorse XF96 Analyzer. Minimal and maximal OCR are also displayed. A two-sample *t*-test was performed and significant values are displayed with asterisks (*p* < 0.005). (**B**) Extracellular acidification rate (ECAR) of *SF3B1*^WT^ (blue) and *SF3B1*^R625G^ (orange) isogenic Mel202 cells as measured by Seahorse XF96 Analyzer. Minimal and maximal ECAR are also plotted. A two-sample *t*-test was performed, but the *p*-values showed no significance (*p* < 0.005).

We found that both minimal and maximal cellular OCR were significantly decreased in *SF3B1*^R625G^ cells as compared to *SF3B1*^WT^ cells in glucose-rich conditions. These results were also confirmed with additional isogenic cell models of *SF3B1*^G742D^-HAP1 and *SF3B1*^K666T^-HEK293T cell lines (Supplementary Figure S6A,B). The decreased respiration can be explained by the underexpression of OXPHOS complexes in *SF3B1*^R625G^ cells (Figure 3A and Figure 4A). Notably, this decrease in OCR was not associated with any significant alterations in ECAR or mitochondrial content (Figure 4B and Figure S5A,C). Taken together, our results indicate that *SF3B1* mutations trigger a decrease in mitochondrial respiration capacity, thus reshaping cell metabolism.

**Figure 5:**
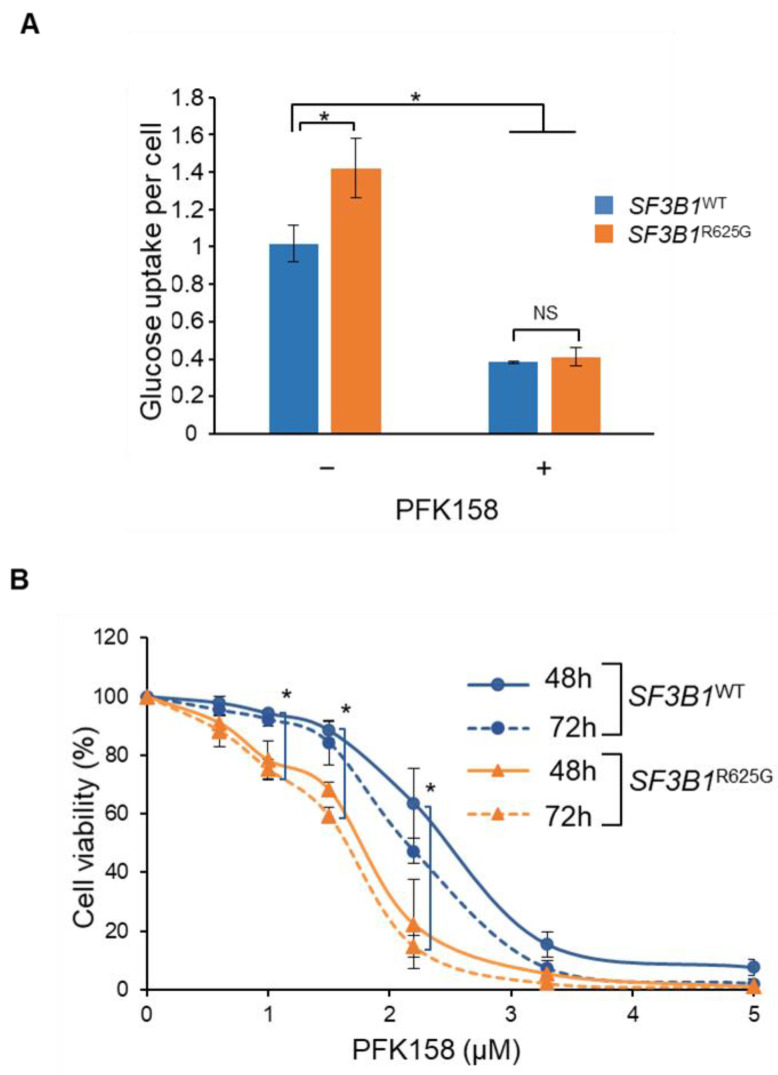
*SF3B1*^R625G^ induces an increase of glycolytic rate, thus sensitizing cells to glycolysis inhibition. (**A**) Glucose uptake rate per cell in *SF3B1*^WT^ (blue) *and SF3B1*^R625G^ (orange) isogenic Mel202 cells with or without PFK158 treatment at 1.6 µM for 3 days. (**B**) Cell viability of *SF3B1*^WT^ (blue) *and SF3B1*^R625G^ (orange) Mel202 cells upon treatment with PFK158 at 48 and 72 h. Data are represented as the mean of triplicates ± SD. The Mann–Whitney U test was used to generate the *p*-values; * *p* < 0.05; NS: non-significant.

We then investigated whether *SF3B1*^R625G^ cells are more dependent on early glycolysis to compensate for decreased mitochondrial respiration. Strikingly, glucose uptake assessment revealed that *SF3B1*^R625G^ cells display an increase of 40% in glucose uptake as compared to *SF3B1*^WT^ cells (Figure 5A). This finding suggests that mutant SF3B1 increases glucose uptake to produce glycolytic intermediates needed to compensate for impaired OXPHOS. To explore this feature, we inhibited the early steps of glycolysis in *SF3B1*^WT^ and *SF3B1*^R625G^ Mel202 cells with PFK158, a PFKFB3 (6-phosphofructo-2-kinase/fructose-2,6-biphosphate 3) inhibitor. As shown in Figure 5A, PFK158 induced a decrease in glucose uptake in both *SF3B1*^WT^ and *SF3B1*^R625G^ Mel202 cells (Figure 5A). Interestingly, *SF3B1*^WT^ cells exhibited IC_50_ values of 2.4 and 2.1 µM at 48 and 72 h of treatment while *SF3B1*^R625G^ cells displayed IC_50_ values of 1.7 and 1.5 µM at 48 and 72 h of treatment, respectively (Figure 5B). Increased sensitivity to PFK158 was also observed in *SF3B1*^G742D^ and *SF3B1*^K666T^ cells as compared to *SF3B1*^WT^ cells (Supplementary Figure S7A,B). These findings indicate that cells mutated for *SF3B1* are more sensitive to glycolysis inhibition than *SF3B1*^WT^ cells.

## 4. Discussion

*SF3B1* missense mutations are recurrent in several cancers, including UM. Mutant SF3B1 corrupts the 3′ ss recognition, leading to the formation of aberrant transcripts. The molecular mechanism by which mutant SF3B1 induces an aberrant splice pattern has been well characterized [7,8,9], yet little is known about its downstream impact and functional involvement. Here, we investigated the fate of aberrant transcripts induced by mutant SF3B1 in UM cells.

Analysis of nascent protein synthesis did not reveal any global impairment in *SF3B1*^R625G^ cells. However, the polysome profiling of *SF3B1*^R625G^ cells revealed target-specific translational alterations. We found that 65% of aberrantly spliced transcripts induced by mutant SF3B1 were less translated than their corresponding canonically spliced transcripts, while 35% were more translated. We focused on the latter group (i.e., 35%), considering that the translated aberrant transcripts are more likely to contribute to cellular dysfunction. Remarkably, this group corresponded to genes enriched for metabolic functions, including carbon metabolism and metabolic pathways. Moreover, quantitative LC-MS/MS analysis showed an altered abundance of metabolic proteins in *SF3B1*^R625G^ cells. Notably, UQCC1 and UQCC2; needed for the assembly of complex III of the mitochondrial respiratory chain, were less abundant in *SF3B1*^R625G^ cells as compared to *SF3B1*^WT^ cells. PHGDH, which is involved in the de novo synthesis of serine, and OXPHOS were also underrepresented. Interestingly, *UQCC1*, *UQCC2* and *PHGDH* are aberrantly spliced by mutant SF3B1, implying that the splicing aberrations of these genes may be at the origin of the protein reduced expression. To explore the impact of such dysregulation of metabolic proteins, we characterized the related metabolic functions in *SF3B1*^R625G^ as compared to *SF3B1*^WT^ Mel202 cells. Our results reveal a reduced rate of mitochondrial respiration in *SF3B1*^R625G^ cells, which is consistent with the observed low abundance of proteins in the electron transport chain.

Given that PHGDH catalyzes the first step of SG synthesis, we evaluated the cell vulnerability to SG starvation and showed that *SF3B1*^R625G^ cells are more dependent on SG than *SF3B1*^WT^ cells. Of note, SG dependency has been reported in cancer and SG deprivation and PHGDH inhibition have been shown to reduce tumor growth [34,35]. Moreover, *SF3B1* hotspot mutations on codons K700, K666 and H662 have been described to induce dysregulation of metabolic enzymes, including PHGDH, in acute myeloid leukemia and breast cancer cells, leading to increased dependency on serine [17,36]. Our study extends these findings to *SF3B1*^R625G^ hotspot mutation in UM cells. Furthermore, based on three different isogenic cell models, our results reveal an increased glucose dependency of *SF3B1*-mutated cells compared to *SF3B1*^WT^ cells. In fact, cells mutated for *SF3B1* display a higher capacity for glucose uptake and are more sensitive to glucose deprivation than *SF3B1*^WT^ cells. This finding suggests that *SF3B1*-mutated cells trigger a metabolic switch toward glycolysis to compensate for their mitochondrial defects, a common feature of tumoral transformation [37]. OXPHOS is the preferable source of ATP for normal cells, while cancer cells have the capacity to switch to early glycolysis. The glycolytic pathway generates ATP and produces metabolic intermediates for cancer cells. Glutamine is also an essential nutrient for cancer cells and exploring its consumption rate when glucose is inhibited can provide further understanding of the process. It will also be worthwhile to assess the impact of SF3B1 inhibitors, including pladienolide B, on metabolism even though specific inhibitors targeting mutant SF3B1 are still lacking [38,39]. Our findings suggest that cells harboring *SF3B1* mutations tend to be addicted to glucose. Accordingly, cells mutated for *SF3B1* display increased sensitivity to PFK158, a glycolysis inhibitor, as compared to *SF3B1*^WT^ cells.

## 5. Conclusions

**Figure 6:**
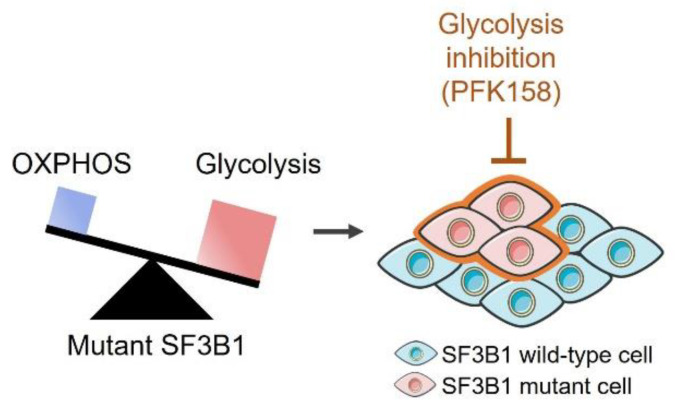
Thematic model of the glycolysis dependency in *SF3B1*-mutated cells.

Overall, we demonstrate that mutant SF3B1 decreases mitochondrial respiration, thereby promoting dependency on glycolysis and sensitizing cells to glycolysis inhibition (Figure 6). Our study sheds light on the oncogenic involvement of mutant SF3B1 through a metabolic switch toward a highly glycolytic rate. These findings offer new therapeutic perspectives for targeting mutant SF3B1 tumors by glycolysis inhibitors or dietary restrictions.

## Data Availability

The data presented in this study are available in this article (and supplementary material).

## Supplementary Materials

The following supporting information can be downloaded at: https://www.mdpi.com/article/10.3390/cancers14092113/s1, Figure S1: Validation of the aberrant splice pattern induced by *SF3B1* mutations in the established isogenic *SF3B1*^WT^ and *SF3B1*^R625G^ Mel202 model; Figure S2: Complete western blots corresponding to Figure 1 and Figure 3; Figure S3: Pathway enrichment of over-represented and under-represented proteins in *SF3B1*^R625G^ cells as compared to *SF3B1*^WT^ cells; Figure S4: Quantifications of replicates of western blots of metabolic proteins; Figure S5: *SF3B1*^WT^ and *SF3B1*^R625G^ cells display comparable ROS levels and equivalent mitochondrial content; Figure S6: *SF3B1*^G742D^ and *SF3B1*^K666T^ cells display a decreased mitochondrial respiration; Figure S7: *SF3B1*^G742D^ and *SF3B1*^K666T^ cells display high sensitivity to glycolysis inhibition; Table S1: Differentially-expressed splice junctions in *SF3B1*^R625G^ versus *SF3B1*^WT^ Mel202 cells; Table S2: Misspliced transcripts in the *SF3B1*^R625G^ Mel202 cells detected in the input, monosomal and polysomal fractions as determined by RNA-seq; Table S3: Gene Ontology enrichment analysis of the translated misspliced transcripts in *SF3B1*^R625G^ Mel202 cells; Table S4: Differentially-abundant proteins in *SF3B1*^R625G^ versus *SF3B1*^WT^ Mel202 cells as determined by LC-MS/MS; Table S5: Gene Ontology enrichment analysis of the differentially-abundant proteins in *SF3B1*^R625G^ versus *SF3B1*^WT^ Mel202 cells.
