# Supplementary material for: Glycolysis Dependency as a Hallmark of SF3B1-Mutated Cells
Source: Cancers (Basel). 2022 Apr 24;14(9):2113. doi: 10.3390/cancers14092113 (PMC9101609; doi:10.3390/cancers14092113)
Supplement: Supplementary file 1 [file cancers-14-02113-s001.zip › Supplementary_Figures.pptx]

## Slide 1
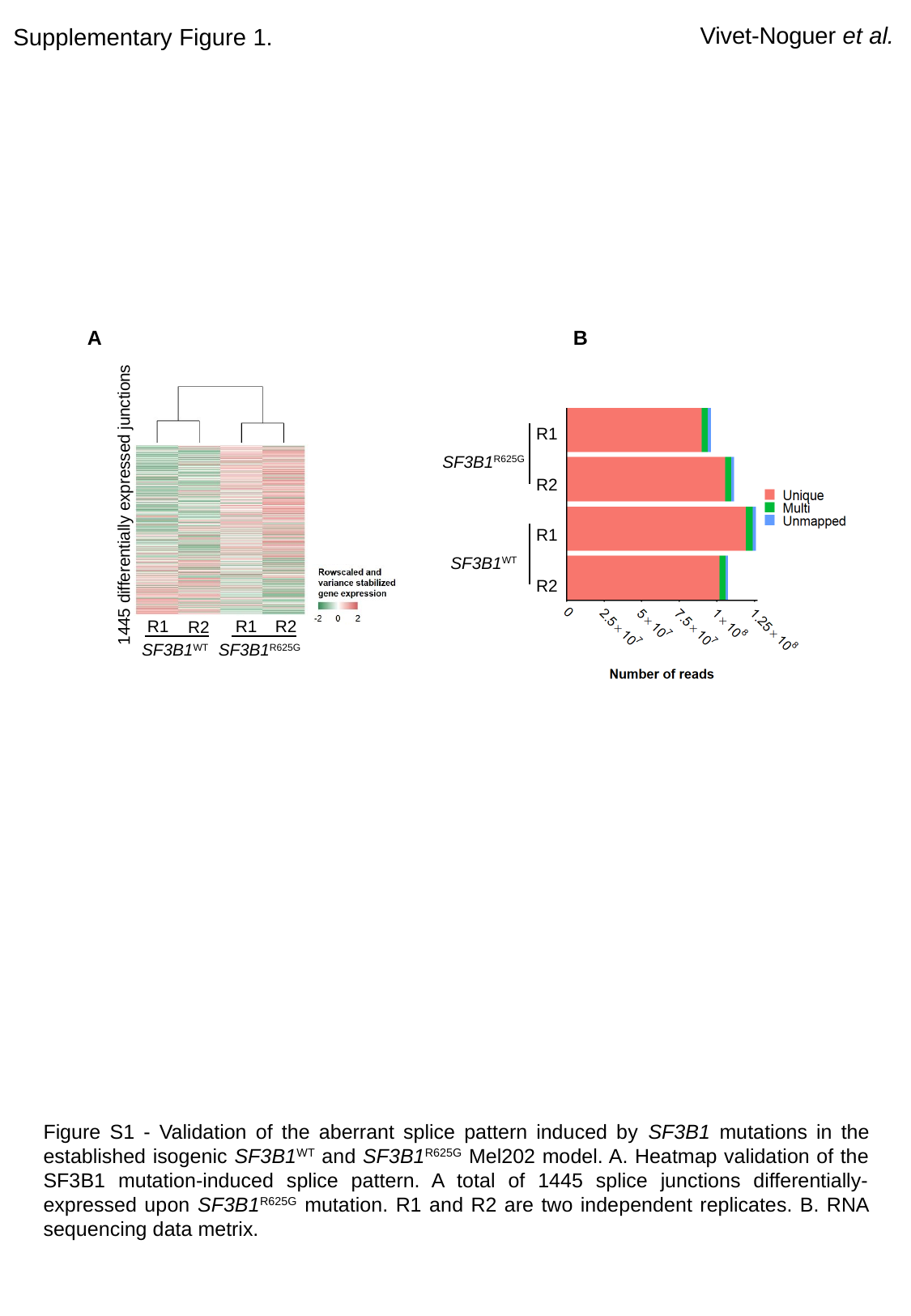

Vivet-Noguer et al.
Supplementary Figure 1.
A				B
R1
R2
R1
R2
SF3B1R625G
SF3B1WT
1445 differentially expressed junctions
R1
R1
R2
R2
SF3B1WT SF3B1R625G
Figure S1 - Validation of the aberrant splice pattern induced by SF3B1 mutations in the established isogenic SF3B1WT and SF3B1R625G Mel202 model. A. Heatmap validation of the SF3B1 mutation-induced splice pattern. A total of 1445 splice junctions differentially-expressed upon SF3B1R625G mutation. R1 and R2 are two independent replicates. B. RNA sequencing data metrix.

## Slide 2
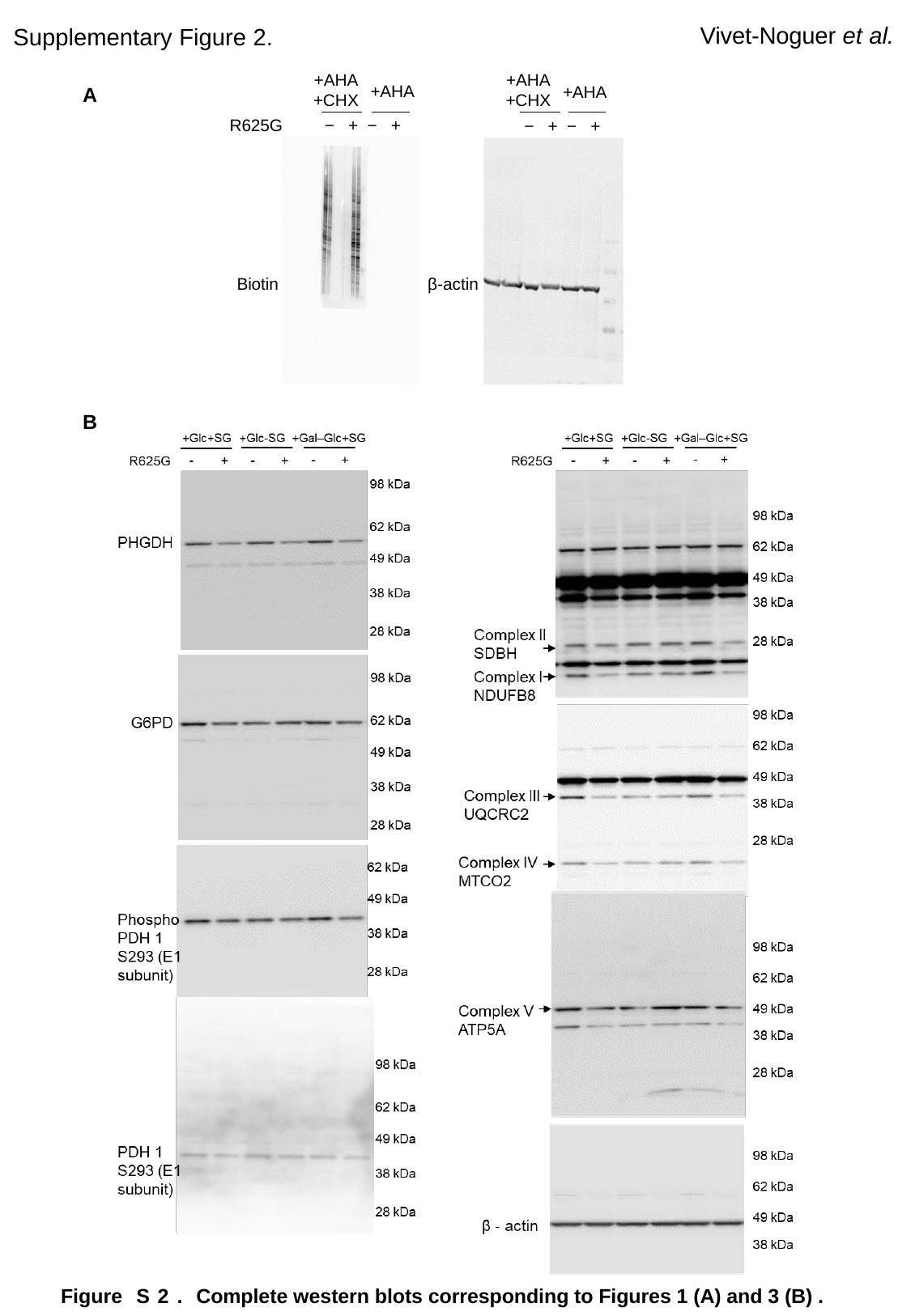

Vivet-Noguer et al.
Supplementary Figure 2.
+AHA
+CHX
+AHA
+CHX
+AHA
+AHA
A
R625G − + − +
 − + − +
Biotin
β-actin
B
Figure S2. Complete western blots corresponding to Figures 1 (A) and 3 (B) .

## Slide 3
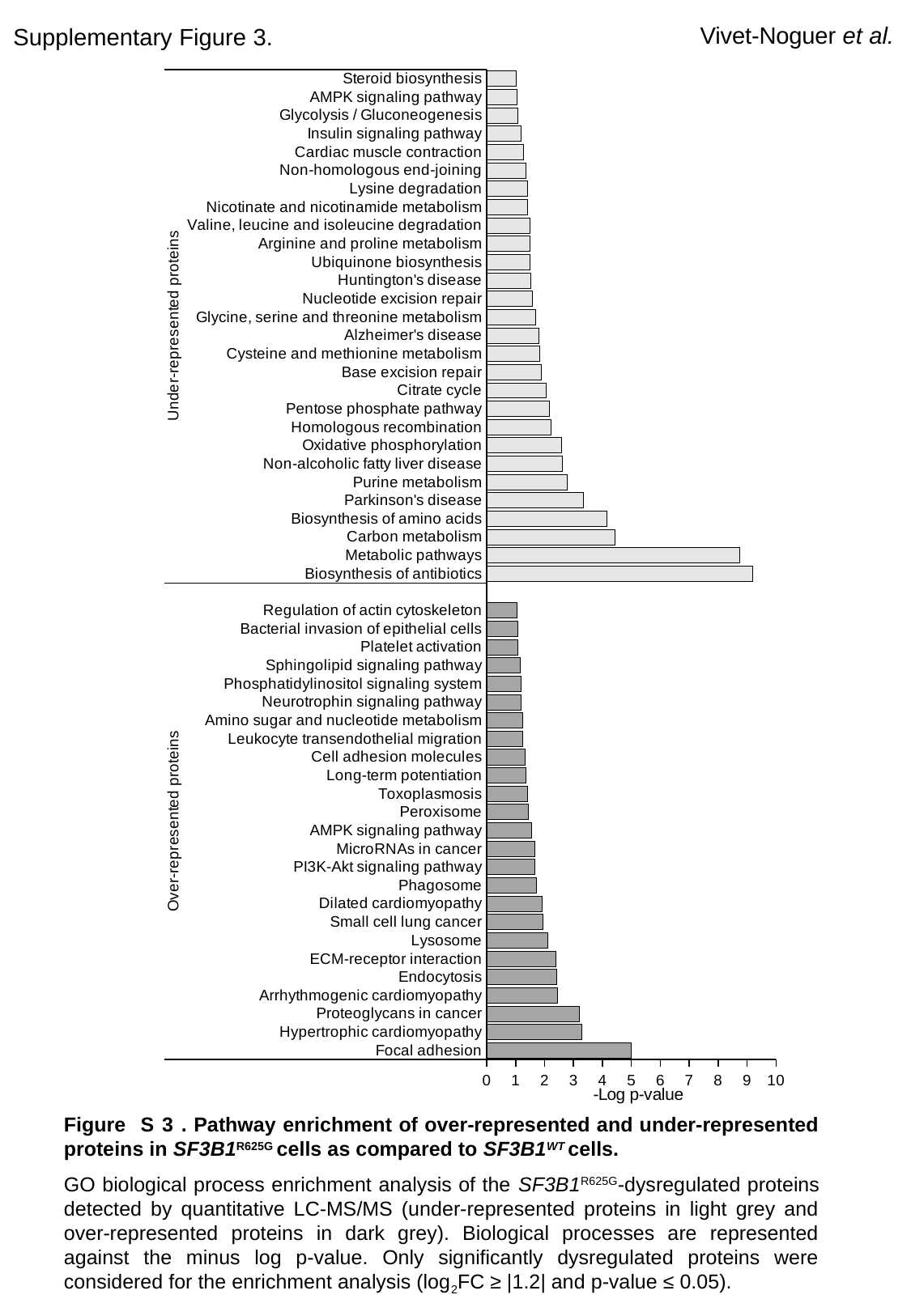

Vivet-Noguer et al.
Supplementary Figure 3.
### Chart
| Category | |
|---|---|
| Focal adhesion | 4.986566820461053 |
| Hypertrophic cardiomyopathy | 3.298906013887644 |
| Proteoglycans in cancer | 3.200198707259038 |
| Arrhythmogenic cardiomyopathy | 2.4579376471520167 |
| Endocytosis | 2.428375569400742 |
| ECM-receptor interaction | 2.390810573278905 |
| Lysosome | 2.1144412107035797 |
| Small cell lung cancer | 1.9510222772915766 |
| Dilated cardiomyopathy | 1.923940664390745 |
| Phagosome | 1.7144486986812735 |
| PI3K-Akt signaling pathway | 1.6627023026454713 |
| MicroRNAs in cancer | 1.659256375655056 |
| AMPK signaling pathway | 1.5530510224972551 |
| Peroxisome | 1.4443534878793862 |
| Toxoplasmosis | 1.4084989604822729 |
| Long-term potentiation | 1.368588822989217 |
| Cell adhesion molecules | 1.330671831714335 |
| Leukocyte transendothelial migration | 1.2510369450154677 |
| Amino sugar and nucleotide metabolism | 1.2496092339077849 |
| Neurotrophin signaling pathway | 1.202890327826369 |
| Phosphatidylinositol signaling system | 1.1888057925974662 |
| Sphingolipid signaling pathway | 1.1718980375668275 |
| Platelet activation | 1.0838952990483013 |
| Bacterial invasion of epithelial cells | 1.0737521426232968 |
| Regulation of actin cytoskeleton | 1.039796425270994 |
| | None |
| Biosynthesis of antibiotics | 9.19889896273169 |
| Metabolic pathways | 8.739763065595273 |
| Carbon metabolism | 4.440829017567412 |
| Biosynthesis of amino acids | 4.16206852063706 |
| Parkinson's disease | 3.334636982915126 |
| Purine metabolism | 2.787678369520593 |
| Non-alcoholic fatty liver disease | 2.6085374660995564 |
| Oxidative phosphorylation | 2.5874639481491304 |
| Homologous recombination | 2.2132543345612588 |
| Pentose phosphate pathway | 2.1597777968100162 |
| Citrate cycle | 2.059107871648752 |
| Base excision repair | 1.8793230077545409 |
| Cysteine and methionine metabolism | 1.8382526606005287 |
| Alzheimer's disease | 1.8001583564940402 |
| Glycine, serine and threonine metabolism | 1.6870024402922543 |
| Nucleotide excision repair | 1.5854489765109139 |
| Huntington's disease | 1.5290188212332092 |
| Ubiquinone biosynthesis | 1.5062018795959968 |
| Arginine and proline metabolism | 1.492517699610958 |
| Valine, leucine and isoleucine degradation | 1.492517699610958 |
| Nicotinate and nicotinamide metabolism | 1.424388053281176 |
| Lysine degradation | 1.4070941515600988 |
| Non-homologous end-joining | 1.3690588745926977 |
| Cardiac muscle contraction | 1.2604743732295296 |
| Insulin signaling pathway | 1.1857017085471715 |
| Glycolysis / Gluconeogenesis | 1.0654189896257729 |
| AMPK signaling pathway | 1.0503100624337824 |
| Steroid biosynthesis | 1.0329531388191553 |Figure S3. Pathway enrichment of over-represented and under-represented proteins in SF3B1R625G cells as compared to SF3B1WT cells.
GO biological process enrichment analysis of the SF3B1R625G-dysregulated proteins detected by quantitative LC-MS/MS (under-represented proteins in light grey and over-represented proteins in dark grey). Biological processes are represented against the minus log p-value. Only significantly dysregulated proteins were considered for the enrichment analysis (log2FC ≥ |1.2| and p-value ≤ 0.05).

## Slide 4
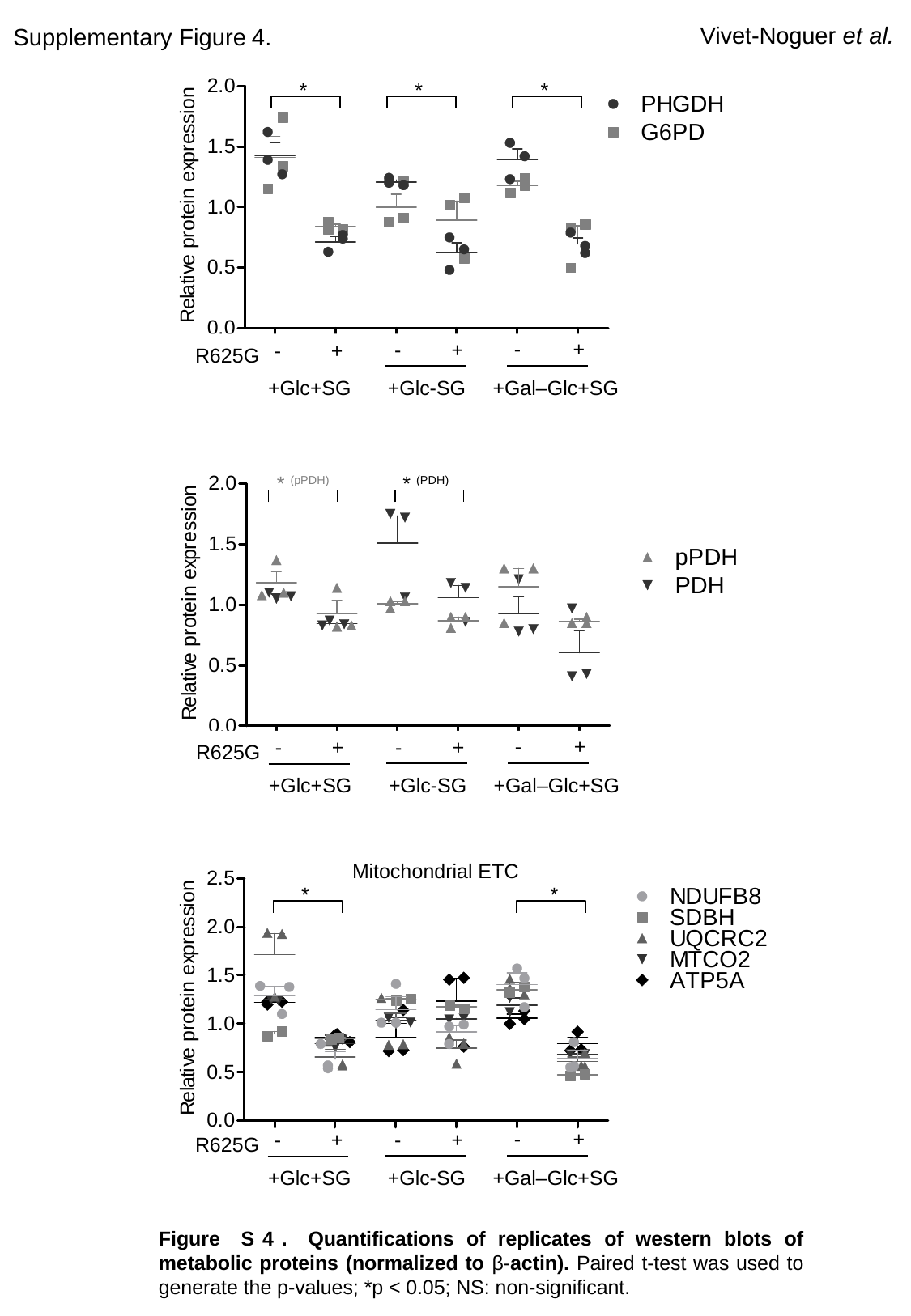

Vivet-Noguer et al.
Supplementary Figure 4.
*
*
*
-
+
-
+
-
+
R625G
+Glc-SG
+Gal–Glc+SG
+Glc+SG
* (pPDH)
* (PDH)
-
+
-
+
-
+
R625G
+Glc-SG
+Gal–Glc+SG
+Glc+SG
Mitochondrial ETC
*
*
-
+
-
+
-
+
R625G
+Glc-SG
+Gal–Glc+SG
+Glc+SG
Figure S4. Quantifications of replicates of western blots of metabolic proteins (normalized to β-actin). Paired t-test was used to generate the p-values; *p < 0.05; NS: non-significant.

## Slide 5
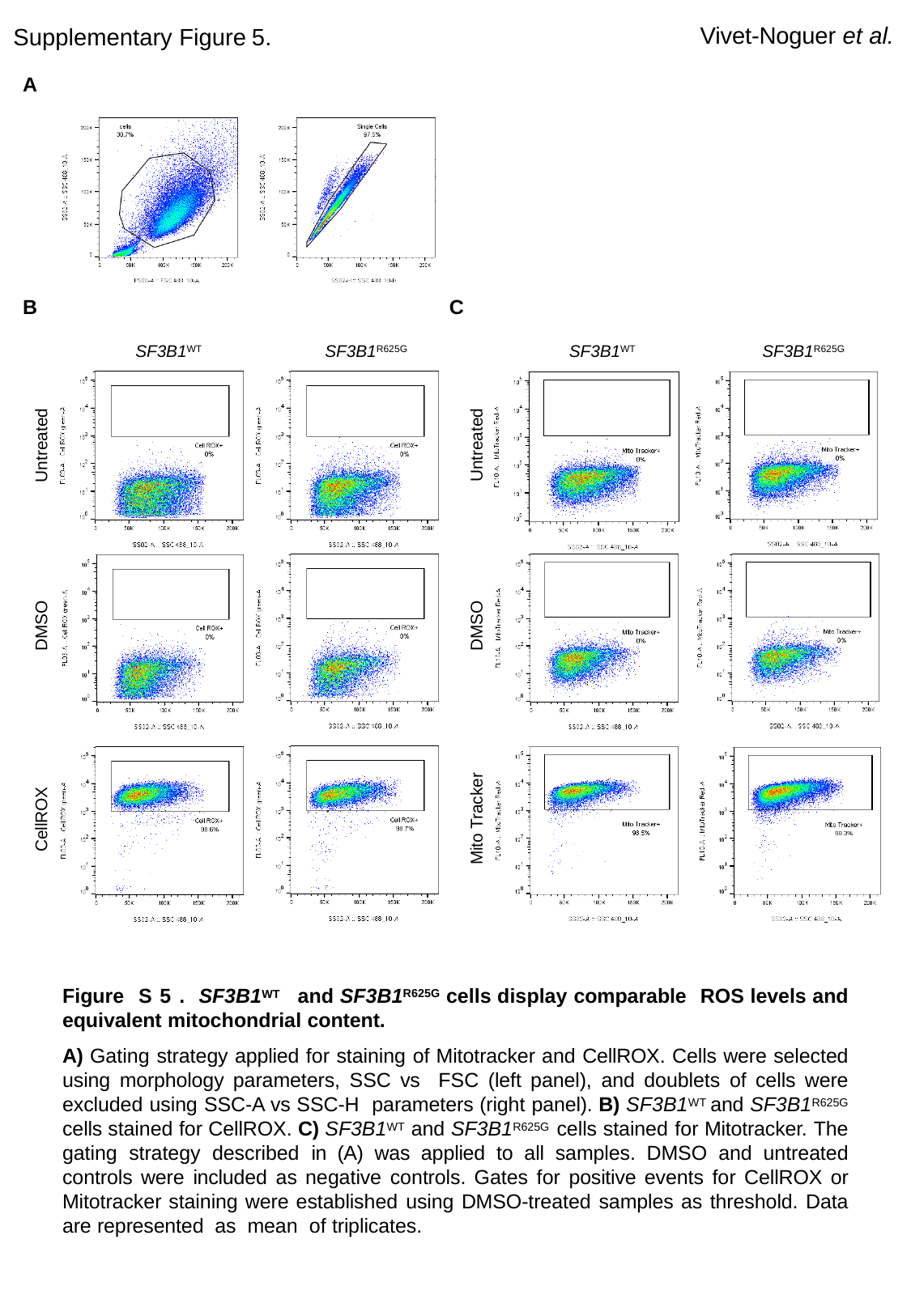

Vivet-Noguer et al.
Supplementary Figure 5.
A
B
C
SF3B1WT
SF3B1R625G
Untreated
DMSO
CellROX
SF3B1WT
SF3B1R625G
Untreated
DMSO
Mito Tracker
Figure S5. SF3B1WT and SF3B1R625G cells display comparable ROS levels and equivalent mitochondrial content.
A) Gating strategy applied for staining of Mitotracker and CellROX. Cells were selected using morphology parameters, SSC vs FSC (left panel), and doublets of cells were excluded using SSC-A vs SSC-H parameters (right panel). B) SF3B1WT and SF3B1R625G cells stained for CellROX. C) SF3B1WT and SF3B1R625G cells stained for Mitotracker. The gating strategy described in (A) was applied to all samples. DMSO and untreated controls were included as negative controls. Gates for positive events for CellROX or Mitotracker staining were established using DMSO-treated samples as threshold. Data are represented as mean of triplicates.

## Slide 6
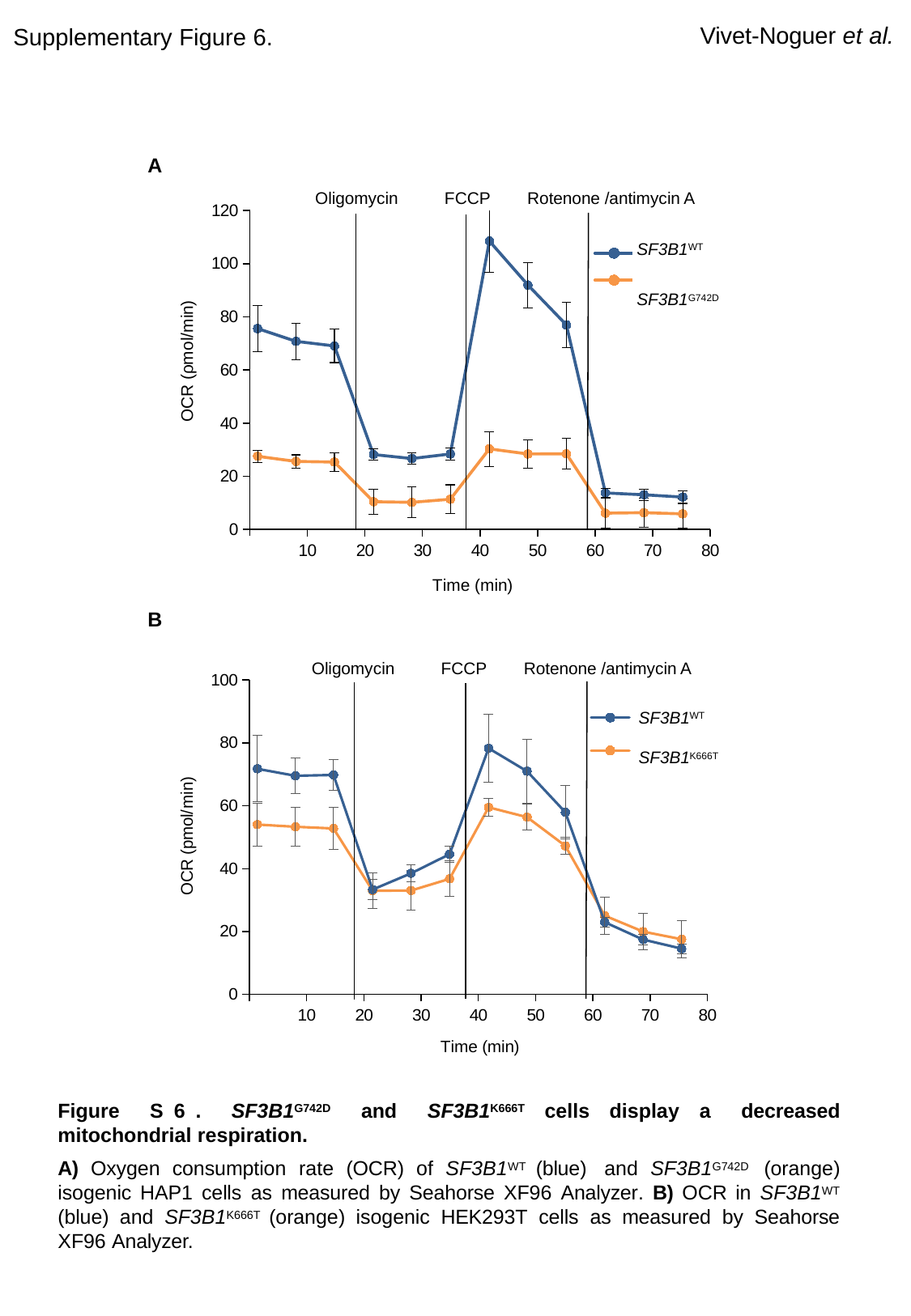

Vivet-Noguer et al.
Supplementary Figure 6.
A
Oligomycin FCCP Rotenone /antimycin A
### Chart
| Category | | |
|---|---|---|OCR (ρmol/min)
SF3B1WT
SF3B1G742D
Time (min)
B
Oligomycin FCCP Rotenone /antimycin A
### Chart
| Category | | |
|---|---|---|SF3B1WT
SF3B1K666T
Figure S6. SF3B1G742D and SF3B1K666T cells display a decreased mitochondrial respiration.
A) Oxygen consumption rate (OCR) of SF3B1WT (blue) and SF3B1G742D (orange) isogenic HAP1 cells as measured by Seahorse XF96 Analyzer. B) OCR in SF3B1WT (blue) and SF3B1K666T (orange) isogenic HEK293T cells as measured by Seahorse XF96 Analyzer.

## Slide 7
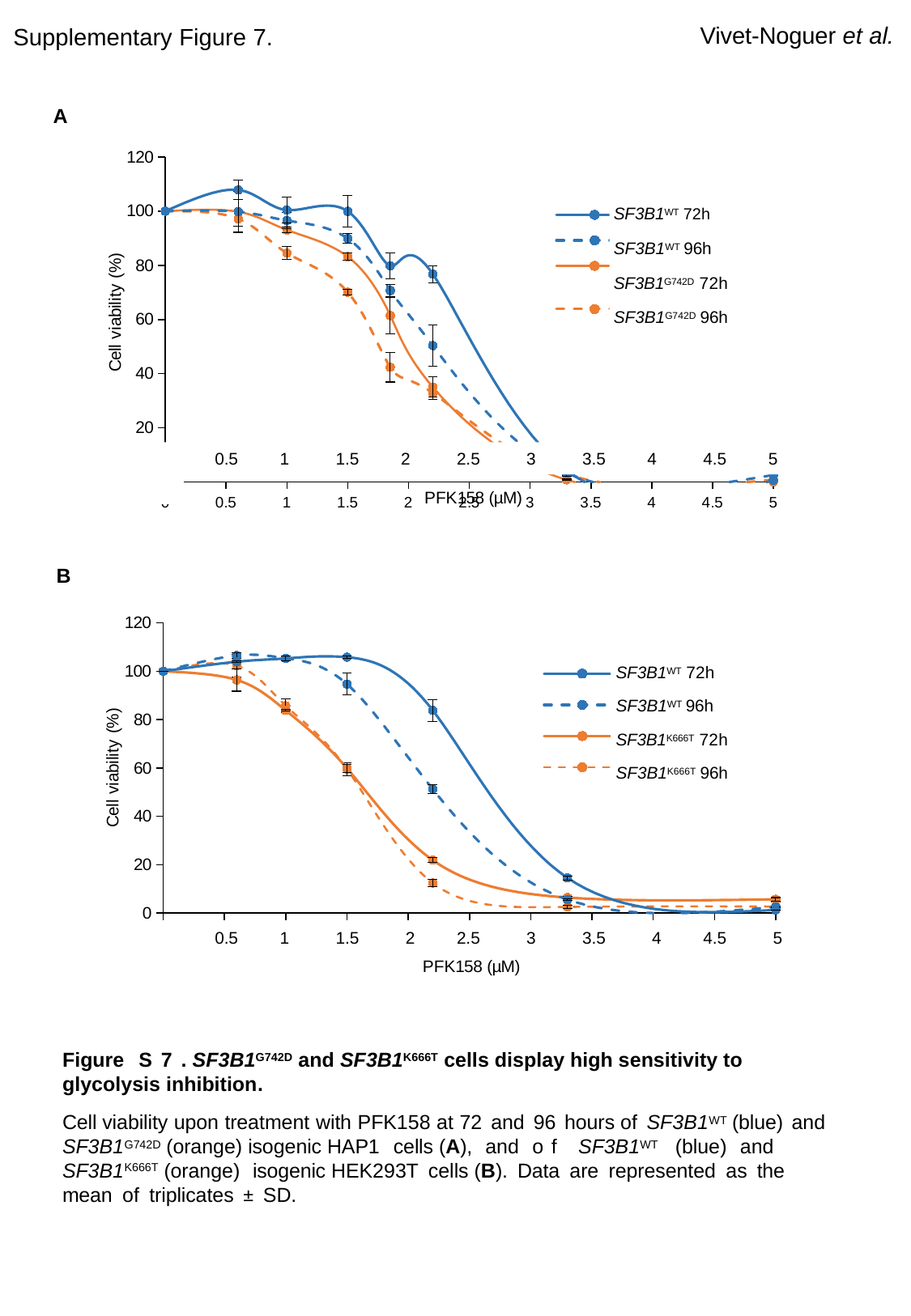

Vivet-Noguer et al.
Supplementary Figure 7.
A
### Chart
| Category | | | | |
|---|---|---|---|---|
SF3B1WT 72h
SF3B1WT 96h SF3B1G742D 72h SF3B1G742D 96h
 0.5 1 1.5 2 2.5 3 3.5 4 4.5 5
B
### Chart
| Category | | | | |
|---|---|---|---|---|
SF3B1WT 72h
SF3B1WT 96h
SF3B1K666T 72h
SF3B1K666T 96h
 0.5 1 1.5 2 2.5 3 3.5 4 4.5 5
Figure S7. SF3B1G742D and SF3B1K666T cells display high sensitivity to glycolysis inhibition.
Cell viability upon treatment with PFK158 at 72 and 96 hours of SF3B1WT (blue) and SF3B1G742D (orange) isogenic HAP1 cells (A), and of SF3B1WT (blue) and SF3B1K666T (orange) isogenic HEK293T cells (B). Data are represented as the mean of triplicates ± SD.
